# Supplementary material for: Empirical methods for controlling false positives and estimating confidence in ChIP-Seq peaks
Source: BMC Bioinformatics. 2008 Dec 5;9:523. doi: 10.1186/1471-2105-9-523 (PMC2628906; doi:10.1186/1471-2105-9-523)
Supplement: Additional File 1 — A variety of html documents from the USeq web site detailing the available applications, their best usage, output file type descriptions, command line menus, etc. [file 1471-2105-9-523-S1.zip › USeqUserGuides/JavaDoc.zip!/JavaDoc/bioroot/antibody/class-use/UseDilution.html]

Uses of Class bioroot.antibody.UseDilution


|  |  |  |
| --- | --- | --- |
| **Overview** \| **Package** \| **Class** \| **Use** \| **Tree** \| **Deprecated** \| **Index** \| **Help** | |  |
| PREV   NEXT | **FRAMES**    **NO FRAMES**     **All Classes** |  |


---


## **Uses of Class bioroot.antibody.UseDilution**

| Packages that use UseDilution | |
| --- | --- |
| **bioroot.antibody** |  |

Uses of UseDilution in bioroot.antibody

| Methods in bioroot.antibody that return UseDilution | |
| --- | --- |
| `UseDilution[]` | `AntibodyPrep.getUseDilutions(DBUtil bioRoot)` |

| Methods in bioroot.antibody with parameters of type UseDilution | |
| --- | --- |
| `static void` | `AntibodyBase.getUseDilutionForm(UseDilution u, java.lang.StringBuffer b, int index, int apbIndex, boolean lastForm)`             Returns a UseDilutionNotes where the names are apended with apbIndex.index ie 0.0, 0.1, 0.2,,1.0,1.1... |
| `static void` | `AntibodyBase.getUseDilutionReport(UseDilution u, java.lang.StringBuffer b, int index, int apbIndex)` |
| `void` | `AntibodyPrep.setUseDilutions(UseDilution[] useDilutions)` |

---


|  |  |  |
| --- | --- | --- |
| **Overview** \| **Package** \| **Class** \| **Use** \| **Tree** \| **Deprecated** \| **Index** \| **Help** | |  |
| PREV   NEXT | **FRAMES**    **NO FRAMES**     **All Classes** |  |


---
